# Pharmacogenomic profile of a central European urban random population-Czech population

**DOI:** 10.1371/journal.pone.0284386

**Published:** 2023-04-20

**Authors:** Riccardo Proietti, Geraldo A. Maranho Neto, Sarka Kunzova, Oriana Lo Re, Ari Ahola-Olli, Juho Heliste, Juan Pablo Gonzalez-Rivas, Manlio Vinciguerra

**Affiliations:** 1 Liverpool Centre for Cardiovascular Sciences (LCCS), University of Liverpool, Liverpool, United Kingdom; 2 International Clinical Research Center, St Anne’s University Hospital, Brno, Czech Republic; 3 Department of Stem Cell Biology and Transplantology, Research Institute of the Medical University of Varna (RIMUV), Varna, Bulgaria; 4 Institute for Molecular Medicine Finland (FIMM), HiLIFE, University of Helsinki, Helsinki, Finland; 5 Stanley Center for Psychiatric Research, The Broad Institute of MIT (Massachusetts Institute of Technology) and Harvard University, Cambridge, MA, United States of America; 6 Institute of Biomedicine, University of Turku, Turku, Finland; 7 Harvard T.H. Chan School of Public Health, Harvard University, Cambridge, MA, United States of America; 8 Liverpool Centre for Cardiovascular Sciences (LCCS), Liverpool John Moores University, Liverpool, United Kingdom; Jordan University of Science and Technology, JORDAN

## Abstract

The genetic basis of variability in drug response is at the core of pharmacogenomics (PGx) studies, aiming at reducing adverse drug reaction (ADR), which have interethnic variability. This study used the Kardiovize Brno 2030 random urban Czech sample population to analyze polymorphisms in a wide spectrum of genes coding for liver enzymes involved in drug metabolism. We aimed at correlating real life drug consumption with pharmacogenomic profile, and at comparing these data with the SUPER-Finland Finnish PGx database. A total of 250 individuals representative of the Kardiovize Brno 2030 cohort were included in an observational study. Blood DNA was extracted and 59 single nucleotide polymorphisms within 13 genes (*BCHE*, *CYP1A2*, *CYP2C9*, *CYP2C19*, *CYP2D6*, *CYP3A5*, *F2*, *F5*, *IFNL3*, *SLCO1B1*, *TPMT*, *UGT1A1*, *VKORC1*), associated to different drug metabolizing rates, were characterized by genotyping using a genome wide commercial array. Widely used drugs such as anti-coagulant warfarin and lipid lowering agent atorvastatin were associated to an alarmingly high percentage of users with intermediate/poor metabolism for them. Significant differences in the frequency of normal/intermediate/poor/ultrarapid/rapid metabolizers were observed for *CYPD26* (p<0.001), *CYP2C19* (p<0.001) and *UGT1A1* (p<0.001) between the Czech and the Finnish study populations. Our study demonstrated that administration of some popular drugs to a Czech random sample population is associated with different drug metabolizing rates and therefore exposing to risk for ADRs. We also highlight interethnic differentiation of some common pharmacogenetics variants between Central (Czech) and North European (Finnish) population studies, suggesting the utility of PGx-informed prescription based on variant genotyping.

## Introduction

Evidence shows the genetic components of inter-individual variability in drug response, which has been linked to ethnicity [1]. However, self-described ethnicity does not predict an individual patient’s genotype or response to medication, becoming a drug prescription based on ethnicity an oversimplification of the multifaceted interplay of ancestry and drug response [2]. Pharmacogenomics (PGx) aims to underlie individual differences in drug use, both in terms of efficacy and toxicity, by defining genetic profiles. Increasing evidence suggests that variants in genes encoding for drug metabolizing enzymes/transporters directly influence their function, which in turn results both into adverse drug reactions (ADRs) and/or altered efficacy [3]. Consequently, most medications are beneficial to a subset of the treated patients, while the remaining ones will either not react to the medications or develop ADRs, which are significant causes of mortality and morbidity [4].

The Kardiovize study is a prospective epidemiological survey of 1% (n = 2160) of the urban population of Brno, the second city of the Czech Republic, a Central European country [5]. The Kardiovize study aimed to determine the presence and burden of cardiovascular risk factors in adults aged 25–64 years and to conduct genetic risk analyses. An advantage of DNA extracted from blood samples of a subset of the Kardiovize participants, representing the prevalence of drug consumption of the overall cohort. We genotyped 59 SNPs within a selected but comprehensive array of genes associated with different drug metabolizing rates. Although some previous studies provided reference frequencies of alleles tested in this study in the Czech Republic or neighboring European countries [6–11], most of them focused on specific diseased patient cohorts and not on the general population. In this study, our goal was to contribute to the definition of a PGx profiling of a general Czech population, in particular for the most commonly used medications. To this purpose, by crossing with validated medicine use from the Kardiovize population surveys, we report here in a general Czech population sample PGx outcomes for widely used widely used drugs (warfarin, atorvastatin and metaprolol). Moreover, by comparing genotyping results to a Finnish reference population (SUPER-Finland study) [12], we investigate significant differences in the PGx metabolic activities between these two European ethnicities.

## Methods

### Study design

The Kardiovize study has been previously described [5]. Recruitment and baseline examinations were completed in 2014 with planned follow-up at 5-year intervals through 2030. The baseline study protocol was approved by the ethics committee of St Anne’s University Hospital, Brno, Czech Republic (reference 2 G/2012), following the Declaration of Helsinki. Written consent was obtained from all participants. Data were stored using the web-based research electronic data capture (REDCap) [13]. For the current analysis we used data from participants with complete anthropometric measurements, sociodemographic and life-style information, and genotype data (subjects with missing genotypes for > 4 SNPs were excluded).

### DNA extraction and sequencing

DNA was extracted from participants’ blood samples using the DNeasy Blood & Tissue Kit from QIAGEN (Germany), according to manufacturer’s instructions. We selected 59 SNPs in 13 loci identified by genome-wide association studies as being associated with different drug metabolizing rates (*BCHE*, *CYP1A2*, *CYP2C19*, *CYP2D6*, *CYP3A5*, *F2*, *F5*, *IFNL3*, *SLCO1B1*, *TPMT*, *UGT1A1*, *VKORC1*). All DNAs were quantified with Nanodrop Lite before any futher processing. For SNP genotyping with OpenArray plate in total 60 ng of sample DNA was loaded to each OpenArray sub-array (Thermo Fisher Scientific^TM^, Vienna, Austria). 2x TaqMan™ OpenArray™ Real-Time PCR Master Mix was used according to manufacturer protocol (Thermo Fisher Scientific^TM^, Vienna, Austria). OpenArray plate preparation was done using AccuFill system according to manufacturer protocol (Thermo Fisher Scientific^TM^, Vienna, Austria). SNP calling was done using TaqMan Genotyper Software (Thermo Fisher Scientific^TM^, Vienna, Austria). Auto-calling function was used and each assay was manually checked. For CNV determination two detection assays were used: *CYP2D6* exon9 in combination with RNAseP and *CYP2D6* intron2 in combination with RNAseP (Thermo Fisher Scientific^TM^, Vienna, Austria). All reactions were carried our according to manufacturer protocol on 384-well plate, each reaction in quadruplicate. In each reaction 10 ng of DNA was used. Real-time PCR run was done in QuantStudio 12K Flex Real-Time PCR System (Thermo Fisher Scientific^TM^, Vienna, Austria). Data analysis was done using CopyCaller® Software (Thermo Fisher Scientific^TM^, Vienna, Austria). For each run a reference sample with known CNV count was used to determine sample calls.

### GeneRx database

The GeneRx database (https://www.generx.fi/) has been developed by Abomics (www.abomics.fi) and includes information about genotypes that are associated with clinically relevant variation in >200 drugs, either considering the responsiveness to the drugs or drug-induced adverse effects. The database is a collection of recommendations for the most clinically relevant and actionable pharmacogenetic drug-gene pairs. The contents of the database reflect published expert opinion pharmacogenetic recommendations and contents of commercial pharmacogenetic test panels. The database is regularly updated based on newly published literature, which is reviewed for each gene-drug pair, and recommendations are changed when needed. Recommendations are mostly based on published expert opinions and pharmacogenetic recommendation articles, e.g. by the Clinical Pharmacogenetics Implementation Consortium (CPIC). The FDA’s list of pharmacogenetic biomarkers in drug labels is also followed for update process.

In the database, predicted phenotypes based on genotypes, considers the four metabolizer types for drug metabolizing enzymes: 1) Poor Metabolizer (PM). Medication is broken down very slowly. May experience side effects at standard doses; 2) Intermediate Metabolizer (IM). Slow rate of metabolism. May be exposed to excessive drug plasma concentration at standard doses, potentially causing side effects; 3) Normal Metabolizer (NM). Normal rate of metabolism. Expected normal efficacy at standard doses; 4) Ultrarapid Metabolizer (UM). Medication is rapidly broken down, potentially leading to lack of efficacy. These descriptions of changes in metabolism are opposite in the case of prodrugs for which the risk of over-exposure and adverse effects is more pronounced in UMs, as lack of efficacy is expected in IMs and PMs. For transporter protein genes (such as *SLCO1B1*), the predicted phenotype classes are increased, normal, decreased, and poor function. For blood coagulation factors F2 and F5, the phenotype stratification considers risk classification for venous thromboembolism (increased or significantly increased risk of venous thromboembolism). IFNL3 phenotypes are associated with the response to antiviral hepatitis C treatment (favorable or unfavorable response genotypes). For *VKORC1*, the phenotypes represent the expression levels of the enzyme (normal or reduced expression), which links it to warfarin sensitivity.

The raw genetic data were interpreted to diplotypes and assigned to predicted phenotypes by Abomics’ in-house interpretation software. The allelic phenotypes are represented in the S1 Table. Matching of diplotypes to phenotypes was done according to CPIC guidelines for CYP2C9, CYP2C19, CYP2D6, CYP3A5, IFNL3, SLCO1B1, TPMT and UGT1A1. For VKORC1, heterozygous carriers of rs9923231 (-1639G>A) were given the phenotype “reduced expression” and homozygous carriers the phenotype “remarkably reduced expression” of the enzyme.

### Statistical analyses

Descriptive statistics were used to summarize the dataset and the distribution of genetic variants. Continuous variables were expressed as mean and standard deviation (SD), and tested for differences with independent-sample *t* tests. Categorical variables were expressed as absolute frequencies and percentages, and tested for differences with chi-squared tests. The distributions of specific SNPs between the Czech and the Finnish populations were compared using the Chi-squared test. Possible deviations from Hardy-Weinberg equlibrium for all detected haplotypes in the Czech population was tested with HardyWeinberg package for R [14] (S1 Table). All other statistical analyses were performed on the SPSS software (version 26.0, SPSS, Chicago, IL, USA), and p-values < 0.05 were considered statistically significant.

## Results

### Characteristics of the study populations

The current analysis was conducted on a subset of a total of 2160 participants with complete health interviews, anthropometric assessment and genotyping, which satisfied inclusion/exclusion criteria [5]. The prevalence of hypertension and dyslipidaemia in this overall cohort was 38.7%, and 67.1%, respectively [5]. Accordingly, Table 1 describes succinctly the medications (by broad classes) taken by the overall baseline population (n = 2160; men = 977, women = 1183).

**Table 1:** Medication frequency in the overall Kardiovize study (n = 2160).

Medications	ATC code PGx
Hypolipidemics	
Fibrates	1.3% C10AB –
Statins	8.2% C10AB CYP2D6
Ezetimibe	0.3% C10AX09 –
Antithrombotics, anticoagulants	
Acetylsalicylic acid	3.1% N02BA01 –
Warfarin	1.0% B01AA03 CYP2C9, VKORC1
Clopidogrel	0.2% B01AC04 CYP2C19
Ticlopidin	0.1% B01AC05 –
Diuretics	
Loop diuretics	1.0% C03 –
Thiazide	4.1% C03 –
Potassium-sparing	1.1% C03 –
Beta, alpha-sympatholytics, Ca blockers, and other vasodilatators	
Beta-adrenergic blockers	8.1% C07 CYP2D6
Alpha-adrenergic blockers	1.0% C07 CYP2D6
Calcium-channel blockers	7.2% C08 –
Angiotensin converting enzyme inhibitors	11.3% C09 –
Sartans	5.0% C09 CYP2C9
Hypoglycemics	
Insulin	1.1% U –
Oral hypoglycemic agents	3.2% A10 CYP2C9

A total of randomly selected 250 participants, aged 25 to 64 years (mean = 47.9 years; SD = 11.3) were included for PGx profiling. No statistically significant indications for deviation from Hardy-Weinberg equilibrium were detected, suggesting a genetically balanced, random sample **(**S1 **Table)**. The prevalence of hypertension, hyperlipidaemia, and diabetes mellitus in this sub-cohort was 38.9% and 71.5%, and 4.4% respectively, similar to the overall cohort. The prevalence of the combinations with either diabetes mellitus and hypertension; or diabetes mellitus and hyperlipidaemia; or hypertension with hyperlipidaemia were respectively 3.6%, 3.2%, and 36.9%.

### Single nucleotide polymorphism (SNPs) in drug metabolizing genes

The list of the 59 SNPs that we detected in 13 drug-metabolizing genes, which have been previously identified by numerous genome-wide association studies as being associated with different drug metabolizing rates [15], is summarized in Table 2. Detected haplotype frequencies, as well as proportions of homozygotes and heterozygotes, are presented in S1 Table. The 13 genes include BCHE (pseudocholinesterase); *CYP1A2*, *CYP2C9*, *CYP2C19*, *CYP2D6*, *CYP3AS* (all members of the cytochrome P450 mixed-function oxidase system family); *F2*, *F5* (coagulation factors); *IFNL3* (interferon Lambda 3); *SLCO1B1* (a solute carrier organic anion transporter family member); *TPMT* (thiopurine methyltransferase); *UGT1A1* (a uridine diphosphate glucuronosyltransferase); and *VKORC1* (a subunit of vitamin K epoxide reductase complex).

**Table 2:** List of 55 single nucleotide polymorphisms (SNPs) or other variants (indel or copy number variation (CNV)), detected in 13 drug metabolizing genes in the Kardiovize study population (n = 250 individuals).

BCHE	CYP1A2	CYP2C9	CYP2C19	CYP2D6	CYP3A5	F2	F5	IFNL3	SLCO1B1	TPMT	UGT1A1	VKORC1
rs1799807	rs12720461	rs1057910	rs12248560	rs1065852	rs10264272	rs1799963	rs6025	rs12979860	rs11045821	rs1142345	rs4148323	rs9923231
rs1803274	rs2069514	rs1799853	rs28399504	rs1135840	rs28365083				rs11045872	rs1800460	rs887829	
rs28933389	rs2069526	rs28371686	rs41291556	rs16947	rs28383479				rs11045879	rs1800462		
rs28933390	rs35694136	rs56165452	rs4244285	rs28371706	rs41303343				rs2306283	rs1800584		
	rs762551		rs4986893	rs28371725	rs55817950				rs4149056			
			rs56337013	rs35742686	rs776746				rs4149081			
			rs72552267	rs3892097								
			rs72558186	rs5030655								
			rs1057910	rs5030865								
			rs1799853	rs5030867								
			rs28371686	rs594213								
			rs56165452	CNV								

### Use of selected widely used drugs among different PGx profile in the Czech population

Subsequently, we focused our attention on the most commonly used medications used by our participants. In our study cohort 8.5% individuals used the anticoagulant warfarin. Warfarin dosage required to reach therapeutic drug levels is heavily affected by two enzymes, CYP2C9 and VKORC1 (Tables 1 and 3).

**Table 3:** Percentage of normal/intermediate/poor metabolizers, or persons with decreased or poor transporter function of SLCO1B1 or reduced expression of VKORC1, showing high proportions of potentially deviating drug responses to warfarin, atorvastatin and metoprolol.

Drug-metabolizing genes	Users	Normal	Intermediate metabolizer / Decreased function / Decreased expression	Poor metabolizer / Poor function / Remarkably decreased expression
**Warfarin**	8.5%			
VKORC1		37.6%	50.4%	12%
CYP2C9		69.5%	26.3%	4.1%
**Atorvastatin**	16.0%			
SLCO1B1		67.5%	30.5%	2%
**Metoprolol**	22.4%			
CYP2D6		84.8%	5.3%	8.2%

CYP2C9 enzyme metabolizes warfarin while VKORC1 (an enzyme involved in vitamin K recycling) is the target of warfarin. A common variant in the *VKORC1* gene (tested in the gene panel) is associated with increased sensitivity to the drug. Only 42.9% of individuals using warfarin exhibited normal expression of *VKORC1*, with 38.1% exhibiting reduced expression and 19% exhibiting markedly reduced expression. Among the warfarin users, 61.9% were normal metabolizers and 38.1% were intermediate metabolizers for *CYP2C9* (Table 3). Genetic analyses of SLCO1B1, a molecular transporter involved in hepatic uptake of statins, showed that atorvastatin users (16% of the total study population) were for 70% with normal function of the transporter, while the remaining 30% of users exhibited decreased function (Table 3). Finally, the utilization of metoprolol by 22% of the study population was associated with a CYP2D6-dependent normal metabolism by 92.8% of individuals, with only 5.3% and 1.9% of individuals displaying intermediate and poor metabolism, respectively (Table 3).

### Comparison between Czech and Finnish PGx profiles

As a comparative ethnically different study population, we considered a Finnish reference population, consisting of 9262 non-related individuals participating in the SUPER-Finland study, previously described [12]. We used the same list of the 59 SNPs that we detected in 13 drug-metabolizing genes, which is summarized in Table 2.

**Fig 1:**
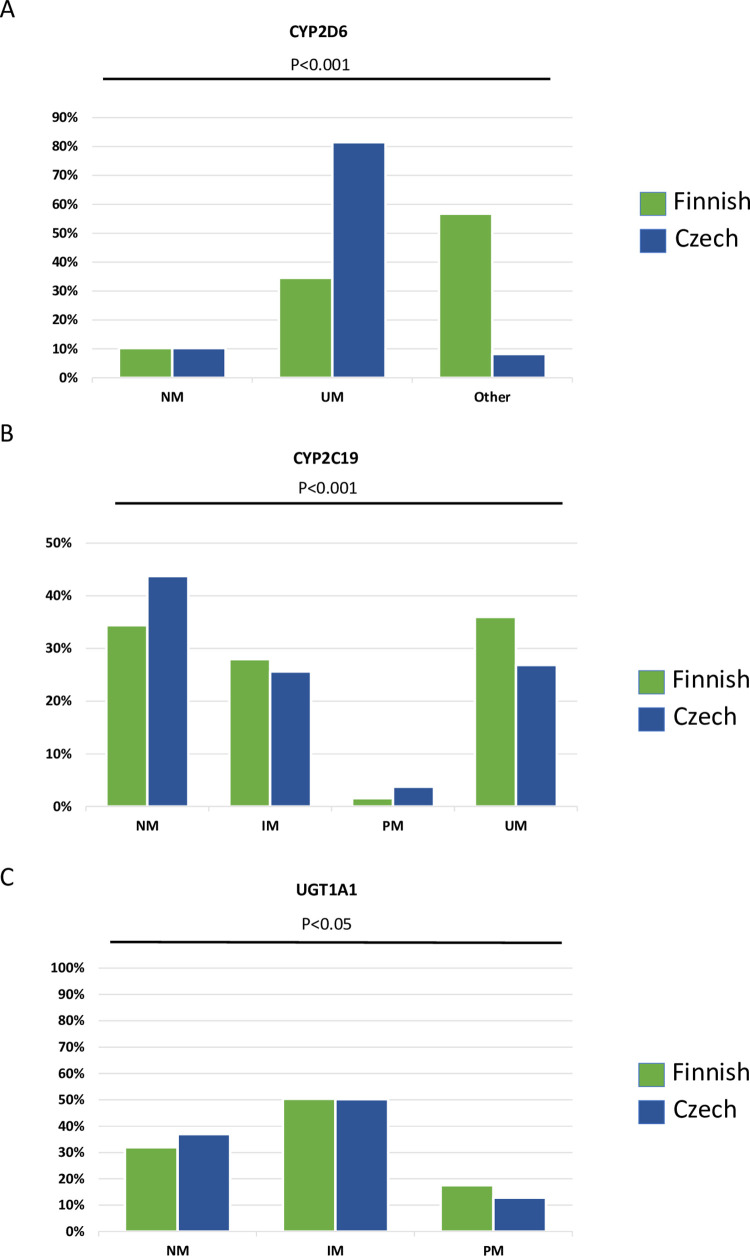
Pharmacogenomics differences between Czech (Kardiovize Brno 2030) and Finnish (SUPER-Finland) reference populations, for the CYP2D6, CYP2C19 and UGT1A1 phenotypes.

We detected significant pharmacogenomics differences in the SNPs specifically for *CYP2D6*, *CYP2C19* and *UGT1A1* between the Czech and the Finnish populations (Fig 1). In particular, for *CYP2D6* the Czech population contained less ultra-rapid metabolizers (UM) (p<0.001); for *CYP2C19* the Czech population contained more normal metabolizers (NM) (~45% versus 40%) and more intermediate metabolizers (IM) (~29% versus ~26%) (p<0.001); for *UGT1A1* the Czech population contained ~37% NM, while Finns included 32% (p<0.001) (Fig 1).

CYP2C19 is a key marker to rationalize clopidogrel treatment, CYP2D6 is responsible for metabolizing anti-depressants and anti-psychotics, while UGT1A1 metabolises many endogenous substances and clinical drugs, such as steroids, bilirubin and irinotecan.

## Discussion

In a Czech random population sample, around 20–30% of patients showed a poor PGx metabolism to warfarin and atorvastatin, exposing them to risk for ADRs. While these data may reflect inter-individual variability rates in the genetic responses to warfarin, atorvastatin and metoprolol that has been previously reported in several European countries [16], at the PGx level it reveals that warfarin and atorvastatin have been prescribed routinely to an alarmingly high number of Czech individuals in Kardiovize cohort who carry actionable pharmacogenetic variants.

Recently PGx has acquired importance to define individual differences in drug efficacy and toxicity, by taking into account the underlying genetic profile [17]. Closely connected to PGx is the discipline of personalized medicine which is linked to the use of an individual’s genetic profile to guide the most suitable therapeutic choice by suggesting predictions about whether that patient will have beneficial effects from a medication or, conversely or suffer important side effects [17]. In several European populations, particularly in those living in countries with lower income, information about the prevalence of pharmacogenomic biomarkers is incomplete or lacking. Studies have shown that ~91% of ADR are type A which are directly related to drug metabolism, hence likely identified by genetic testing [18].

The clinical relevance of our findings showing a high prevalence of impaired metabolism in association with warfarin treatment is consistent with prior work highlighting an increased risk of bleeding in patients carrying variants for *CYP2C9* [19]. Accordingly, the GIFT study has reported that genotype-guided warfarin dosing in the range of therapeutic international normalized ratio, improves outcomes such as bleeding, and death [20]. In addition to CYP2C9, other similar enzymes such as CYP2C8 serve as minor pathways to metabolize warfarin. Interestingly, it has been shown that genetic variations of the gene encoding the CYP2C8 drug metabolizing enzymes can lead to clinical differences in drug metabolism and ultimately variations in drug effectiveness and toxicities within populations in the same country. This is observed in different populations living in Jordan [21], which include Chechens (~1%) and Circassians, genetically isolated groups [22, 23]. Chechens display several pharmacogenomics variants (i.e *ABCB1*, *VDR*) resembling those present in Europeans and Finnish [24]. Our Kardiovize study was conducted in South Moravia region (Brno) of Czech Republic. In a similar fashion, future studies should assess differences in these important pharmacogene polymorphisms also in a larger Czech population representative of Czech (~65%), Moravian (~5%) and Slovak (~1%) ethnicities. PGx (SNPs) variants may impact on the structure and the activity of the protein/enzyme, and they are predicted to produce normally functioning, or less functioning, enzymes upon transcription/translation, as it has been described among others for CYP2D6 [25], CYP2C19 [26] and UGT1A1 [27]. An additional finding of our study is to highlight interethnic differences of the latter common pharmacogenetics variants (*CYP2D6*, *CYP2C19* and *UGT1A1*) between Czech and Finnish population studies, suggesting the utility of PGx-informed prescription based on variant genotyping. For *CYP2C19*, our data are consistent with the differences observed in the prevalence of high-risk genotypes in a large study assessing the pharmacogenomic biomarkers allelic spectrum in 18 European populations by analyzing 1,931 pharmacogenomics biomarkers in 231 genes [16]. The latter was a pan-European PGx biomarkers spectrum including Croatian, Czech, Dutch, German, Greek, Hungarian, Maltese, Polish, Serbian, Slovenian, Turkish, Cypriot, Italian, Lithuanian, Russian, Slovakian, Spanish and Ukrainian, but not Finnish [16]. Other reports also demonstrated that the Finnish pharmacogenome is rather distinct from non-Finnish Europeans [28–30]. Our data show that Czech individuals are more NM and less RM for *CYP2D6* (metabolizing psychoactive medications such as SSRI, tricyclic antidepressants) compared to Finnish individuals. In this respect, according to the SUPER-Finland study, based on the imputation of 9262 individuals, there is a higher frequency of *CYP2D6* UM in Finland compared with non-Finnish Europeans [12]. The Finns have been shown to have a high frequency of *CYP2D6* UM phenotypes compared with the ancestral European population [31]. A recent pan-European survey comprising 258,888 individuals demonstrated that the prevalence of depression in Finns doubles the one in Czechs [32]. *CYP2D6* genotype had a substantial clinical effect on anti-psychotics exposure and on their therapeutic failure [33]. Pre-emptive *CYP2D6* genotyping would be valuable for personalizing anti-depressant and anti-psychotics dosing and treatment.

UGT1A1 metabolizes chemotherapeutic drugs such as irinotecan, used against colorectal cancer. Our data show decreased NM in Finns compared to Czechs; the SUPER-Finland showed a 22-fold enrichment of the *UGT1A1* decreased function variant rs4148323 (UGT1A1*6) in Finland compared with non-Finnish Europeans from the GnomAD v2.1.1 database [12, 34]. Interestingly, the decrease in colorectal cancer mortality in Finland, as monitored by a retrospective analysis of the WHO mortality database, is one of the lowest in Europe despite the constant improvements in screening programs and detection [35]. The World Health Organization’s (WHO) European region includes 53 states with diverse sociopolitical and economic backgrounds. In general, comparisons among countries can help to identify opportunities for the reduction of inequalities in health managements and outcomes. For instance, by comparing two urban population-based samples from Central Europe (Czech and Swiss) we found that increasing age and being male were the main determinants of poor metabolic health independent of obesity status [36].

In terms of CVD, the first cause of death worldwide, the Central and Easter European (CEE) countries have the highest CVD mortality in the EU, which also occur at younger ages [37]. The European region is considered “a natural epidemiologic laboratory” that, due to its enormous diversity, can provide useful lessons [38]. Innovative solutions must be sought to improve the cardiovascular, mental and health in CEE countries. These efforts should take into consideration not only the local context, idiosyncrasy, traditions, social factors and equity implications [39], but also genetic variability. Although few pharmacogenetic tests have been implemented as the standard of care in health systems worldwide, pharmacogenetic evidence-based approaches that rigorously interrogates whether a genetic test genuinely improves the quality of care in a cost-effective and country-specific manner is warranted.

## Conclusion

Our PGx study on the Kardiovize Brno 2030 database shows heterogeneity in the metabolic profile of the population for the most common widely used medications. Of note by crossing with validated medicine we showed that in a relevant proportion the some widely used medications, i.e. warfarin and atorvastatin, are administered to patients with high-risk genotypes exposing them at risk for ADR. Finally comparing our findings in the Czech population with a Finnish reference population we showed interethnic differences in some common pharmacogenetics variants among ethnically different populations worldwide. Our results may contribute to facilitate European integration of PGx and to support pre-emptive PGx testing.

## Supporting information

S1 Table Allelic phenotypes of the raw genetic data. (XLSX)
